# Supplementary figures and images for: Levodopa ONOFF-state freezing of gait: Defining the gait and non-motor phenotype
Source: PLoS One. 2022 Jun 2;17(6):e0269227. doi: 10.1371/journal.pone.0269227 (PMC9162361; doi:10.1371/journal.pone.0269227)

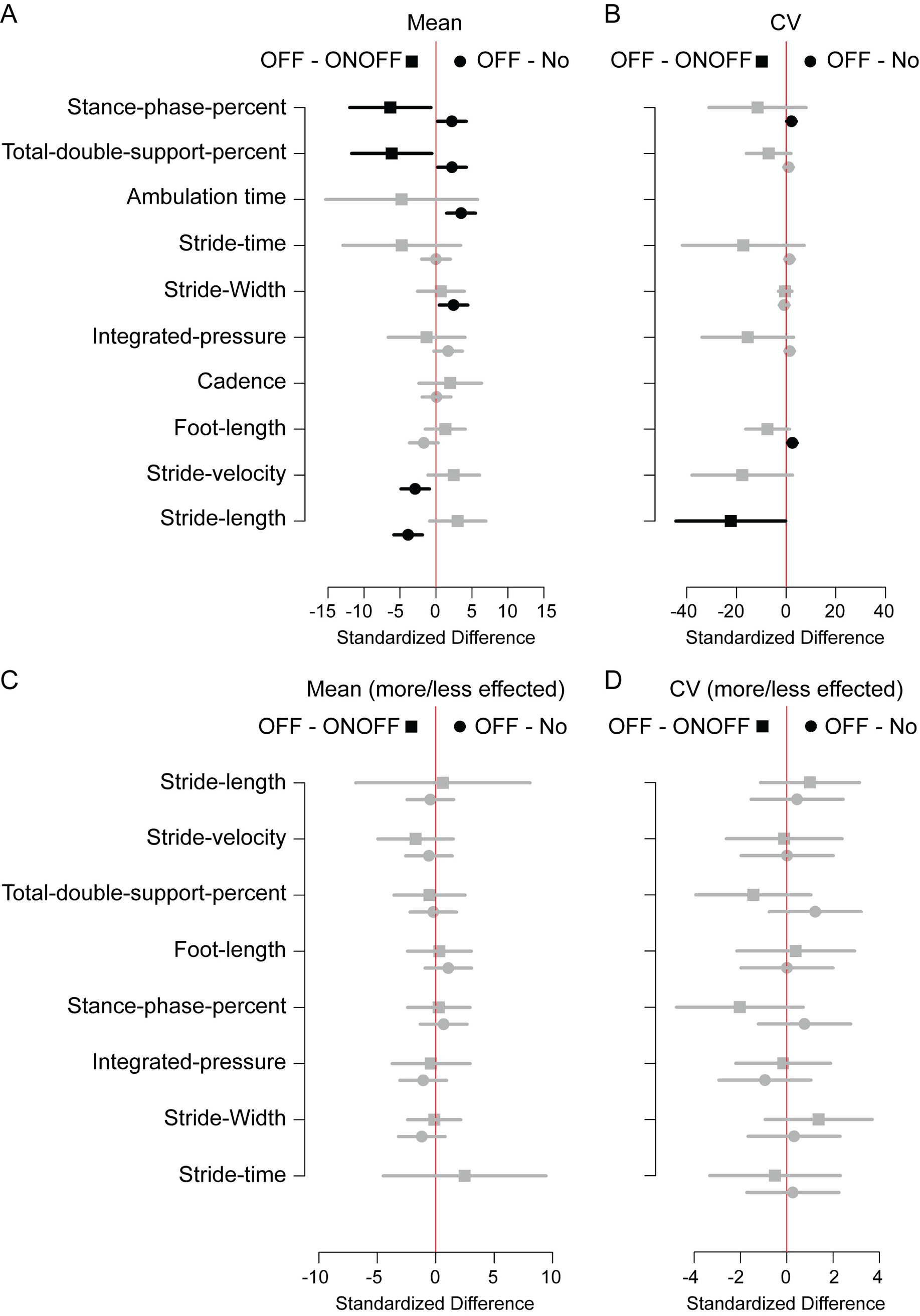

Supplement: S1 Fig — The standardized difference between OFF-state freezers and ON-state freezers (OFF-ON, squares) and OFF-state freezers and non-freezers (OFF-No, circles) is shown for (A) mean and (B) stride-to-stride variability (CV). The standardized differences in the asymmetry ratio (more/less affected side) are also shown for (C) mean and (D) CV. Bars denote the 95% confidence intervals. Comparisons with significant group differences are represented by black symbols and bars, while those without significant group differences are represented with gray symbols and bars. (TIF) [file pone.0269227.s001.tif]
