# Supplementary material for: Levodopa ONOFF-state freezing of gait: Defining the gait and non-motor phenotype
Source: PLoS One. 2022 Jun 2;17(6):e0269227. doi: 10.1371/journal.pone.0269227 (PMC9162361; doi:10.1371/journal.pone.0269227)
Supplement: S1 Table — (PDF) [file pone.0269227.s002.pdf]

S1 Table. Original and standardized results.

VARTYPE	FEATURE	OFF_FOG	_DXGRP	MEAN2	DIFF	LOWER95	UPPER95	TVALUE	DF	PROBT	STANDARDIZER	stOFF_FOG	stMEAN2	stDIFF	stLOWER95	stUPPER95
CV	FtLnth	3.763813904	ON-FOG	4.573099663	-0.809285759	-2.334840711	-2.334840711	-1.081903799	31.02109381	0.287634151	0.268609286	14.01222555	17.02509889	-3.012873344	-8.69233057	2.666583882
CV	FtLnth	3.763813904	noFOG	3.075588547	0.688225357	0.149425389	0.149425389	2.56218006	52.84048897	0.013289319	0.268609286	14.01222555	11.45004549	2.56218006	0.556292713	4.568067407
CV	IntPrs	11.32778998	ON-FOG	13.09153675	-1.763746776	-4.316659772	-4.316659772	-1.399262319	37.46464373	0.169959723	0.665291104	17.02681715	19.67790742	-2.651090275	-6.488377413	1.186196863
CV	IntPrs	11.32778998	noFOG	10.43508555	0.892704423	-0.433041818	-0.433041818	1.341825281	73.58318912	0.183778787	0.665291104	17.02681715	15.68499187	1.341825281	-0.650905769	3.334556332
CV	StLnth	5.691792503	ON-FOG	8.397946442	-2.70615394	-5.77961517	-5.77961517	-1.81042223	25.83937248	0.081875062	0.348314581	16.34095388	24.11023512	-7.769281239	-16.59308994	1.054527462
CV	StLnth	5.691792503	noFOG													
CV	StTime	3.553040239	ON-FOG	5.422220103	-1.869179864	-3.932917571	-3.932917571	-1.857167458	27.38692787	0.074072652	0.299545684	11.86143026	18.10147966	-6.240049398	-13.12960855	0.649509753
CV	StTime	3.553040239	noFOG	3.184122533	0.368917706	-0.228740724	-0.228740724	1.231590791	68.4788669	0.222314677	0.299545684	11.86143026	10.62983947	1.231590791	-0.763625504	3.226807087
CV	StVel	7.207741828	ON-FOG	9.322582192	-2.114840363	-5.07356513	-5.07356513	-1.464288907	27.94648552	0.154274284	0.435325861	16.5571184	21.41518118	-4.858062778	-11.65463755	1.938511994
CV	StVel	7.207741828	noFOG													
CV	StWdth	25.51381225	ON-FOG	29.45287451	-3.939062267	-20.55669713	-20.55669713	-0.47655029	48.20660174	0.635835454	5.856462118	4.356523056	5.029124055	-0.672600998	-3.510087953	2.164885956
CV	StWdth	25.51381225	noFOG	30.15664583	-4.642833587	-16.30782791	-16.30782791	-0.792771044	75.6634314	0.430390052	5.856462118	4.356523056	5.1492941	-0.792771044	-2.784586937	1.199044849
CV	StncPct	2.84497813	ON-FOG	3.151068207	-0.306090077	-1.280224097	-1.280224097	-0.632926614	44.84640122	0.529995926	0.268877494	10.58094557	11.71934534	-1.13839977	-4.761365772	2.484566231
CV	StncPct	2.84497813	noFOG	2.256259311	0.588718819	0.05025451	0.05025451	2.189542941	56.77419236	0.032682628	0.268877494	10.58094557	8.391402632	2.189542941	0.186904859	4.192181022
CV	TotDSupPct	5.477414577	ON-FOG	6.04582463	-0.568410053	-1.664102603	-1.664102603	-1.048121583	40.4300879	0.300808125	0.316637296	17.29870313	19.09384875	-1.795145615	-5.255548307	1.665257078
CV	TotDSupPct	5.477414577	noFOG	5.110448467	0.36696611	-0.263790106	-0.263790106	1.158947841	75.12806077	0.250147952	0.316637296	17.29870313	16.13975529	1.158947841	-0.833098657	3.150994339
CVRatio	FtLnth	1.102057115	ON-FOG	0.99693719	0.105119925	-0.080922135	-0.080922135	1.131248506	57.49432572	0.262647143	0.079290132	13.89904502	12.57328201	1.325763015	-1.020582671	3.672108702
CVRatio	FtLnth	1.102057115	noFOG	1.121971441	-0.019914326	-0.177767916	-0.177767916	-0.251157687	78.02940013	0.802352429	0.079290132	13.89904502	14.15020271	-0.251157687	-2.24199294	1.739677567
CVRatio	IntPrs	0.999690139	ON-FOG	1.003175528	-0.003485389	-0.151956874	-0.151956874	-0.047107336	51.95267962	0.962608302	0.070156507	14.24942874	14.29910893	-0.049680195	-2.165969803	2.066609414
CVRatio	IntPrs	0.999690139	noFOG	1.067498712	-0.067808573	-0.207477428	-0.207477428	-0.966532917	78.07169205	0.336762154	0.070156507	14.24942874	15.21596166	-0.966532917	-2.957351191	1.024285357
CVRatio	StLnth	1.029810401	ON-FOG	0.966085763	0.063724638	-0.0578061	-0.0578061	1.049388354	58.55595848	0.29831134	0.055506721	18.55289568	17.40484303	1.14805265	-1.04142524	3.33753054
CVRatio	StLnth	1.029810401	noFOG	1.007097898	0.022712504	-0.087772655	-0.087772655	0.409184748	78.91758793	0.683512496	0.055506721	18.55289568	18.14371093	0.409184748	-1.581297782	2.399667278
CVRatio	StTime	0.99561427	ON-FOG	1.012612758	-0.016998488	-0.146546019	-0.146546019	-0.263405956	51.14183318	0.793296106	0.044493796	22.37647407	22.75851583	-0.382041763	-3.293628164	2.529544639
CVRatio	StTime	0.99561427	noFOG	0.984906664	0.010707606	-0.077907395	-0.077907395	0.240653901	76.11013199	0.810470437	0.044493796	22.37647407	22.13582017	0.240653901	-1.750972103	2.232279905
CVRatio	StVel	0.986403727	ON-FOG	0.989409709	-0.003005982	-0.108086875	-0.108086875	-0.057237838	59.16612191	0.954548605	0.040730332	24.21791527	24.29171732	-0.073802055	-2.65371948	2.50611537
CVRatio	StVel	0.986403727	noFOG	0.985920801	0.000482926	-0.080715863	-0.080715863	0.011856677	71.77457273	0.990572857	0.040730332	24.21791527	24.20605859	0.011856677	-1.981713863	2.005427217
CVRatio	StWdth	1.054390122	ON-FOG	0.967240866	0.087149256	-0.054661863	-0.054661863	1.230277004	57.70841719	0.223584961	0.059506981	17.71876342	16.25424187	1.464521549	-0.918578992	3.84762209
CVRatio	StWdth	1.054390122	noFOG	1.036858858	0.017531265	-0.100953121	-0.100953121	0.294608539	77.37449388	0.76908192	0.059506981	17.71876342	17.42415488	0.294608539	-1.696492063	2.28570914
CVRatio	StncPct	1.049082583	ON-FOG	1.190746851	-0.141664268	-0.330803351	-0.330803351	-1.500845298	55.28624352	0.139085446	0.066934031	15.67338125	17.78985724	-2.116475983	-4.942229649	0.709277683
CVRatio	StncPct	1.049082583	noFOG	0.996391705	0.052690877	-0.080780616	-0.080780616	0.787206097	70.73009824	0.433791184	0.066934031	15.67338125	14.88617516	0.787206097	-1.206869135	2.781281328
CVRatio	TotDSupPct	1.025854897	ON-FOG	1.092094052	-0.066239155	-0.185451282	-0.185451282	-1.117500312	47.49159984	0.269401939	0.04618677	22.21101213	23.64517078	-1.434158651	-4.015246888	1.146929587
CVRatio	TotDSupPct	1.025854897	noFOG	0.968928048	0.056926849	-0.035028898	-0.035028898	1.232535846	77.73423535	0.221463803	0.04618677	22.21101213	20.97847628	1.232535846	-0.758418438	3.22349013
Mean	AmbTime	39.80835951	ON-FOG	38.86602349	0.942336023	-5.952045583	-5.952045583	0.274519053	50.10524238	0.784814481	1.984042039	20.06427219	19.5893145	0.47495769	-2.999959409	3.949874789
Mean	AmbTime	39.80835951	noFOG	33.04587141	6.762488099	2.786229233	2.786229233	3.408439925	54.90798937	0.001230306	1.984042039	20.06427219	16.65583227	3.408439925	1.404319656	5.412560194
Mean	Cadence	106.7593802	ON-FOG	105.1124506	1.646929574	-7.550762001	-7.550762001	0.361280891	42.29568457	0.719687953	2.397596474	44.52766816	43.84075959	0.686908574	-3.149304765	4.523121912
Mean	Cadence	106.7593802	noFOG	106.3618383	0.397541851	-4.388196121	-4.388196121	0.16580849	66.91224044	0.868807639	2.397596474	44.52766816	44.36185967	0.16580849	-1.830247987	2.161864967
Mean	FtLnth	30.36667029	ON-FOG	30.04945806	0.317212227	-0.873428618	-0.873428618	0.533188235	58.57887827	0.595920489	0.432134053	70.27141248	69.5373527	0.734059779	-2.021198312	3.489317869
Mean	FtLnth	30.36667029	noFOG	31.05982368	-0.693153396	-1.555386409	-1.555386409	-1.604024009	68.33731883	0.113320898	0.432134053	70.27141248	71.87543649	-1.604024009	-3.599314606	0.391266588
Mean	IntPrs	160.6932071	ON-FOG	150.1985303	10.4946768	-12.70984544	-12.70984544	0.906353749	55.03452599	0.368699918	8.554225424	18.78524345	17.55840218	1.226841272	-1.485797347	3.939479892
Mean	IntPrs	160.6932071	noFOG	146.4735881	14.21961903	-2.815969756	-2.815969756	1.66229183	76.45168867	0.100549367	8.554225424	18.78524345	17.12295162	1.66229183	-0.329190501	3.653774161
Mean	StLnth	109.8158943	ON-FOG	104.0963056	5.719588769	-5.826086034	-5.826086034	0.996675372	46.85173272	0.324042011	3.361761417	32.66617725	30.9648106	1.701366653	-1.73304566	5.135778966
Mean	StLnth	109.8158943	noFOG	122.4822364	-12.66634206	-19.37440158	-19.37440158	-3.767769479	68.1295416	0.000346214	3.361761417	32.66617725	36.43394673	-3.767769479	-5.763169711	-1.772369246
Mean	StTime	1.13219496	ON-FOG	1.159997681	-0.027802721	-0.130140628	-0.130140628	-0.548607704	41.12955396	0.586240595	0.025866851	43.77011248	44.84495231	-1.074839826	-5.031174055	2.881494403
Mean	StTime	1.13219496	noFOG	1.132535801	-0.000340841	-0.051985933	-0.051985933	-0.013176743	65.9822928	0.989526546	0.025866851	43.77011248	43.78328923	-0.013176743	-2.009751163	1.983397677
Mean	StVel	98.28510785	ON-FOG	92.68967986	5.595427991	-7.424321473	-7.424321473	0.863635032	49.02099103	0.391994486	3.917496958	25.08875154	23.66043442	1.428317125	-1.895169684	4.751803934
Mean	StVel	98.28510785	noFOG	109.2199098	-10.93480195	-18.75494537	-18.75494537	-2.791272608	66.63529303	0.006842696	3.917496958	25.08875154	27.88002415	-2.791272608	-4.787481794	-0.795063421
Mean	StWdth	13.27446449	ON-FOG	12.1963256	1.07813889	-1.232155363	-1.232155363	0.93656247	51.70790546	0.353337493	0.733151862	18.10602301	16.63546971	1.470553299	-1.680627748	4.621734346
Mean	StWdth	13.27446449	noFOG	11.55951485	1.714949635	0.251788518	0.251788518	2.339146531	67.54274963	0.022293695	0.733151862	18.10602301	15.76687648	2.339146531	0.343432966	4.334860097
Mean	StncPct	66.39028514	ON-FOG	68.39519728	-2.004912139	-4.101235023	-4.101235023	-1.937457204	37.21771627	0.060306166	0.469600919	141.3759694	145.645365	-4.269395688	-8.733447607	0.19465623
Mean	StncPct	66.39028514	noFOG	65.40883263	0.981452511	0.043765606	0.043765606	2.089971444	65.63093904	0.040499098	0.469600919	141.3759694	139.2859979	2.089971444	0.093197445	4.086745444
Mean	TotDSupPct	32.68404301	ON-FOG	36.49835353	-3.814310524	-7.93416533	-7.93416533	-1.874769562	37.6864317	0.06859037	0.936624304	34.89557432	38.96797614	-4.072401824	-8.471022262	0.326218614
Mean	TotDSupPct	32.68404301	noFOG	30.70920031	1.974842696	0.10455533	0.10455533	2.108468345	65.51839491	0.038818631	0.936624304	34.89557432	32.78710598	2.108468345	0.111629956	4.105306734
MnRatio	FtLnth	0.997419423	ON-FOG	0.995251631	0.002167793	-0.016579442	-0.016579442	0.231541909	57.09677692	0.817721634	0.006638094	150.2569111	149.9303426	0.326568567	-2.497621044	3.150758178
MnRatio	FtLnth	0.997419423	noFOG	0.990264382	0.007155042	-0.006103217	-0.006103217	1.077875975	64.72447927	0.285089978	0.006638094	150.2569111	149.1790352	1.077875975	-0.919423113	3.075175063
MnRatio	IntPrs	0.986056591	ON-FOG	1.004517351	-0.01846076	-0.073840905	-0.073840905	-0.671091651	45.74589153	0.505536743	0.016870012	58.45025868	59.54455305	-1.094294374	-4.377050994	2.188462246

VARTYPE	FEATURE	OFF_FOG	_DXGRP	MEAN2	DIFF	LOWER95	UPPER95	TVALUE	DF	PROBT	STANDARDIZER	stOFF_FOG	stMEAN2	stDIFF	stLOWER95	stUPPER95
MnRatio	IntPrs	0.986056591	noFOG	1.002974768	-0.016918177	-0.050564748	-0.050564748	-1.002855015	69.95360899	0.319387249	0.016870012	58.45025868	59.4531137	-1.002855015	-2.99731538	0.991605351
MnRatio	StLnth	0.999528831	ON-FOG	1.004274292	-0.00474546	-0.011269346	-0.011269346	-1.465798561	44.18706291	0.149783613	0.001793711	557.2408517	559.8864625	-2.645610786	-6.282700405	0.991478833
MnRatio	StLnth	0.999528831	noFOG	1.000324853	-0.000796022	-0.004373887	-0.004373887	-0.44378495	69.53345178	0.658576021	0.001793711	557.2408517	557.6846366	-0.44378495	-2.43845736	1.550887459
MnRatio	StTime	0.99969618	ON-FOG	0.999750289	-0.00005411	-0.005769347	-0.005769347	-0.019232631	34.37996201	0.984766714	0.001209799	826.3326952	826.3774213	-0.044726066	-4.768849058	4.679396927
MnRatio	StTime	0.99969618	noFOG													
MnRatio	StVel	1.000013039	ON-FOG	1.005083485	-0.005070446	-0.012129	-0.012129	-1.442310424	50.73709094	0.155359813	0.002191713	456.2700475	458.5835099	-2.313462427	-5.534027123	0.907102269
MnRatio	StVel	1.000013039	noFOG	1.001073565	-0.001060526	-0.005433855	-0.005433855	-0.483880018	68.14308669	0.630022454	0.002191713	456.2700475	456.7539275	-0.483880018	-2.479273083	1.511513046
MnRatio	StWdth	0.995659464	ON-FOG	0.996485743	-0.000826279	-0.040488599	-0.040488599	-0.041828016	50.78375534	0.966799824	0.016849371	59.09178897	59.14082814	-0.04903917	-2.402973959	2.304895619
MnRatio	StWdth	0.995659464	noFOG	1.015950341	-0.020290877	-0.053828713	-0.053828713	-1.204251345	78.99974917	0.232088131	0.016849371	59.09178897	60.29604031	-1.204251345	-3.194701654	0.786198963
MnRatio	StncPct	0.997021854	ON-FOG	0.993717581	0.003304273	-0.015982623	-0.015982623	0.342228582	64.26102478	0.733295673	0.007061664	141.1879591	140.7200421	0.467917015	-2.263294299	3.199128329
MnRatio	StncPct	0.997021854	noFOG	0.992476551	0.004545303	-0.009612039	-0.009612039	0.643658919	54.07349937	0.522517309	0.007061664	141.1879591	140.5443002	0.643658919	-1.36115796	2.648475797
MnRatio	TotDSupPct	0.99885156	ON-FOG	1.000799894	-0.001948335	-0.007366168	-0.007366168	-0.7244098	44.75861279	0.47258407	0.001788234	558.5687827	559.6583128	-1.089530126	-4.119242053	1.940181801
MnRatio	TotDSupPct	0.99885156	noFOG	0.999069118	-0.000217558	-0.003778209	-0.003778209	-0.121661014	77.24159034	0.903483445	0.001788234	558.5687827	558.6904437	-0.121661014	-2.112816022	1.869493994

Supplementary Table 1 - Key

**Column la**	**Description**
VARTYPE	Indicates whether measure is mean (Mean), %CV (CV), ratio of means (MnRatio), or ratio of CVs (CVRatio)
FEATURE	Indicates the gait kinematic parameter: stride-length (StLnth), stride-width (StWdth), stride-time (StTime),
	stride-velocity (StVel), stance-phase-percent (StncPct), total-double-support-percent (TotalDSupPct),
	integrated-pressure applied during a step (IntPrs), foot-strike-length (FtLnth), cadence and ambulation time (AmbTime).
OFF_FOG	Mean for OFF_FOG estimated by the linear mixed model
_DXGRP	Indicates the FOG group that is compared to OFF_FOG
MEAN2	Mean of the indicated _DXGRP estimated by the linear mixed model
DIFF	The difference in means, computed as OFF_FOG - DIFF
LOWER95	Lower bound of the 95% confidence interval for the true difference
UPPER95	Upper bound of the 95% confidence interval for the true difference
TVALUE	Value of the t-statistic comparing the two FOG groups
DF	Degrees of freedom for the t-statistic, estimated with the Kenward & Roger method (2009)
PROBT	p-value testing the mean difference is 0
STANDAR	Value used to standardize the original means (OFF_FOG, MEAN2, DIFF).
	The standardizing value is the standard error of a difference between OFF_FOG and no-FOG.
stOFF_FO	Standardized OFF_FOG mean
stMEAN2	Standardized MEAN2
stDIFF	Standardized DIFF
stLOWER9	Standardized LOWER95
stUPPER9	Standardized UPPER95

SAS code used to produce the results are available from Reid D. Landes
Email rdlandes@uams.edu or rdlandes@gmail.com
