# Supplementary material for: Levodopa ONOFF-state freezing of gait: Defining the gait and non-motor phenotype
Source: PLoS One. 2022 Jun 2;17(6):e0269227. doi: 10.1371/journal.pone.0269227 (PMC9162361; doi:10.1371/journal.pone.0269227)
Supplement: S1 File — (DOCX) [file pone.0269227.s004.docx]

**Supplementary methods:**

**Statistical analysis:**

For each feature within each measure type {mean, CV, ratio of diseased and dominant means, ratio of diseased and dominant CVs}, we used a linear mixed model that accounted for FOG group, motor UPDRS, and the interaction of FOG group and motor UPDRS, sex, age, and disease duration. The initial model was as follows:

| $Y_{jk}=\begin{matrix} \beta_{Off}+{\beta_{No}NoFOG}_{jk}+\beta_{On}{OnFOG}_{jk} \\ + \beta_{Off*M}M_{jk}+\beta_{No*M}{NoFOG}_{jk}\times M_{jk}+\beta_{On*M}{OnFOG}_{jk}\times M_{jk} \\ + \beta_{sex}{MALE}_{jk}+\beta_{age}{AGE}_{jk}+\beta_{D}{DURATION}_{jk}+\varepsilon_{jk} \end{matrix}$ | (1) |
| --- | --- |

where

- $Y_{jk}$ is the feature measure from patient $k$in group $j\in\left\{ \text{No-FOG, Off-FOG, On-FOG} \right\}$,
- $\beta_{Off}$ is the mean of the Off-FOG group, and is the reference mean,
- $\beta_{No}$ is the mean difference in the No-FOG group from the Off-FOG group,
- ${NoFOG}_{jk}$ is an indicator variable for whether patient $k$ in group $j$ is in the No-FOG group, taking value 1 for $j$ = No-FOG, and 0 otherwise,
- $\beta_{On}$ is the mean difference in the On-FOG group from the Off-FOG group,
- ${OnFOG}_{jk}$ is an indicator variable for whether patient $k$ in group $j$ is in the On-FOG group, taking value 1 for $j$ = On-FOG, and 0 otherwise,
- $\beta_{Off*M}$ is the slope on Motor UPDRS score, $M$, for the Off-FOG group, and is the reference slope,
- $\beta_{No*M}$ is the difference in the slope on $M$ of the No-FOG group from the Off-FOG group’s slope,
- $\beta_{On*M}$ is the difference in the slope on $M$ of the On-FOG group from the Off-FOG group’s slope, $\beta_{sex}$ is the difference of males from females (females as reference), and ${MALE}_{k}$ is an indicator variable of male sex for patient $k$; note all patients were cisgender.
- $\beta_{age}$ is the expected change in $Y$ for 10 years’ increase in age,
- $\beta_{D}$ is the expected change in $Y$ for a year’s increase in disease duration, and
- $\varepsilon_{jk}$ is the random error associated with patient $k$ in group $j$, and is assumed to be independently distributed normal with mean 0 and variance, $\sigma_{j}^{2}$, dependent upon group $j$.

Note, we centered $AGE$ about the mean age of all 105 patients, 67.2 years at enrollment, so that FOG group means would be at 67.2 years of age rather than 0 years of age. Similarly, we centered $DURATION$ about the mean disease duration of 8.3 years at enrollment, and centered $M$ about the mean motor UPDRS score of 17.17 at enrollment.

If we could not reject the null hypothesis that $\beta_{Off*M}= \beta_{No*M}=\beta_{On*M}$ at the 0.10 significance level, we refitted the model with a common slope on $M$, namely,

| $Y_{k}=\begin{matrix} \beta_{Off}+{\beta_{No}NoFOG}_{jk}+\beta_{On}{OnFOG}_{jk} \\ + \beta_{M}M_{jk} \\ + \beta_{sex}{MALE}_{jk}+\beta_{age}{AGE}_{jk}+\beta_{D}{DURATION}_{jk}+\varepsilon_{jk} \end{matrix}$ | (2) |
| --- | --- |

where $\beta_{M}$ is the slope on $M$, assumed the same among the 3 FOG groups.

If there was no evidence of a difference among $\beta_{Off*M}= \beta_{No*M}=\beta_{On*M}$ at the 0.10 significance level, then we estimated the difference of On-FOG from Off-FOG and of No-FOG from Off-FOG. We note these differences do not depend on the value of *M*. Figures 1, 2, and Supplementary Figure 1 show the differences along with 95% confidence intervals for those differences for all the features we analyzed. In order to concisely visualize these differences from 8 to 10 features within a measure type, all having different scales of measurement, we standardized the differences (see the supplementary file with original and standardized results for the standardizing value).

If there was evidence of a difference among $\beta_{Off*M}= \beta_{No*M}=\beta_{On*M}$, we determined which of $\beta_{No*M}$ and $\beta_{No*M}$ differed from $\beta_{Off*M}$. If only $\beta_{No*M}$ differed from $\beta_{Off*M}$, then we fitted the model

| $Y_{jk}=\begin{matrix} \beta_{Off}+{\beta_{No}NoFOG}_{jk}+\beta_{On}{OnFOG}_{jk} \\ + \beta_{M}M_{jk}+\beta_{No*M}{NoFOG}_{jk}\times M_{jk} \\ + \beta_{sex}{MALE}_{jk}+\beta_{age}{AGE}_{jk}+\beta_{D}{DURATION}_{jk}+\varepsilon_{jk} \end{matrix}$ | (3A) |
| --- | --- |

where $\beta_{M}$ is the slope on $M$, assumed the same between the Off-FOG and On-FOG groups, and $\beta_{No*M}$ is the difference of the No-FOG slope on $M$ from $\beta_{M}$. And if only $\beta_{On*M}$ differed from $\beta_{Off*M}$ then we fitted the model

| $Y_{jk}=\begin{matrix} \beta_{Off}+{\beta_{No}NoFOG}_{jk}+\beta_{On}{OnFOG}_{jk} \\ + \beta_{M}M_{jk}+\beta_{On*M}{OnFOG}_{jk}\times M_{jk} \\ + \beta_{sex}{MALE}_{jk}+\beta_{age}{AGE}_{jk}+\beta_{D}{DURATION}_{jk}+\varepsilon_{jk} \end{matrix}$ | (3B) |
| --- | --- |

where $\beta_{M}$ is the slope on $M$, assumed the same between the Off-FOG and No-FOG groups and $\beta_{On*M}$ is the difference of the On-FOG slope on $M$ from $\beta_{M}$.

We note that only 3 features showed evidence of a difference among $\beta_{Off*M}= \beta_{No*M}=\beta_{On*M}$, and then only the slope of $M$ for NoFOG patients, $\beta_{No*M}$, was found to differ from the other two, $\beta_{Off*M}$ and $\beta_{On*M}$. That is, we used model 3A for these 3 features: CVs of stride length, CVs of stride velocity, and ratios of diseased to dominant means of stride time. For these 3 features, the difference of On-FOG from Off-FOG did not depend on $M$, thus these differences appear in Figures 2 and 3. Since the difference of No-FOG from Off-FOG depended on $M$, we compared these 2 groups at low, middle, and high values of $M$.

**Power analysis:**

For the power analysis presented in the Discussion, we assumed a modified version of the model in Equation 2 above; namely,

| $Y_{k}=\begin{matrix} \beta_{Off}+\beta_{On}{OnFOG}_{jk} \\ + \beta_{M}M_{jk}+ \beta_{sex}{MALE}_{jk}+\beta_{age}{AGE}_{jk}+\beta_{D}{DURATION}_{jk}+\varepsilon_{jk} \end{matrix}$ | (2) |
| --- | --- |

where

- $Y_{jk}$ is the feature measure from patient $k$in group $j\in\left\{ \text{Off-FOG, On-FOG} \right\}$,
- $\beta_{Off}$ is the mean of the Off-FOG group, and is the reference mean,
- $\beta_{On}$ is the mean difference in the On-FOG group from the Off-FOG group,
- ${OnFOG}_{jk}$ is an indicator variable for whether patient $k$ in group $j$ is in the On-FOG group, taking value 1 for $j$ = On-FOG, and 0 otherwise,
- $\beta_{M}$ is the slope on Motor UPDRS score, $M$, assumed the same between the Off-FOG and On-FOG groups,
- $\beta_{sex}$ is the difference of males from females (females as reference), and ${MALE}_{k}$ is an indicator variable of male sex for patient $k$,
- $\beta_{age}$ is the expected change in $Y$ for a year increase in age of onset,
- $\beta_{D}$ is the expected change in $Y$ for a year’s increase in disease duration, and
- $\varepsilon_{jk}$ is the random error associated with patient $k$ in group $j$, and is assumed to be independently distributed normal with mean 0 and variance, $\sigma_{j}^{2}$, dependent upon group $j$.

The parameter of interest is $\beta_{On}$. We estimated the power for detecting values of $\beta_{On}$ ranging from ½ to 1½ in increments of ¼; the units on these are $\sigma_{Off}$ – the population standard deviation of Off-FOG patients. The parameters $\beta_{M}$, $\beta_{sex}$, $\beta_{age}$, and $\beta_{D}$ are nuisance parameters in that their values do not affect the power on detecting non-zero $\beta_{On}$; we set these all equal to 0.01. Similarly, the value $\beta_{Off}$ does not affect $\beta_{On}$ power; we set $\beta_{Off}$ equal to 0. Finally, and importantly, we assumed $\sigma_{On}$ was double that of $\sigma_{Off}$; i.e., $\sigma_{On}=2\sigma_{Off}$.

Because age at onset, disease duration ($D$), and the score from UPDRS Part III ($M$) are random variables, we randomly generated values for these from normal distributions. Age at onset was generated with a standard deviation of 10 years, disease duration with a standard deviation of 5 years, and $M$ with a standard deviation of 15 for Off-FOG patients and 20 for On-FOG patients. We assumed the sample was exactly half female and half male. For each of the 5 values of $\beta_{On}$, we generated 1,000 data sets having equal numbers of Off-FOG and On-FOG patients, with total sample sizes ranging from 20 to 200 in increments of 10. Then, for a given $\beta_{On}$ and sample size, we computed power on the 1,000 datasets, and averaged those power values to plot against sample size in Figure 4. Datasets and power calculations were performed with custom code using in SAS/STAT software, version 9.4 (SAS System for Windows, SAS Institute, Inc.); the code is Chunk #600 in the supplementary file “Landes et al - Analyses (03FEB2022).sas”.
