# Supplementary material for: Levodopa ONOFF-state freezing of gait: Defining the gait and non-motor phenotype
Source: PLoS One. 2022 Jun 2;17(6):e0269227. doi: 10.1371/journal.pone.0269227 (PMC9162361; doi:10.1371/journal.pone.0269227)
Supplement: Attachment — Submitted filename: Landes et al - Response to Reviewers v2.docx [file pone.0269227.s007.docx]

Response to Reviewers:

We thank the reviewers for taking the time to review our manuscript and provide constructive criticisms. We have extensively modified the manuscript and individually responded to each reviewer comment below in the blue indented text. We believe that our manuscript is improved by these comments and we hope are now acceptable for publication.

Reviewer #1: This manuscript aims to characterize persons with Parkinson’s disease (PD) who experience freezing of gait (FoG) while on parkinsonian medications and contrast these characteristics against those of persons with PD who either only experience FoG when off of their medications or do not experience FoG at all. The authors report that persons with PD + FoG when medicated had worsened disease severity, cognition, quality of life, and global gait characteristics than participants in the other groups. I found the topic of the study to be interesting; however, the reporting of the results is challenging to follow given the qualitative nature with which the results are reported and interpreted. The introduction also lacks important background that could help the reader to better understand the motivations behind the study.

Response: We thank the reviewer for these suggestions. We have expanded on the introduction as requested. We have also clarified why statistical tests were not performed for baseline characteristics, and identified statistically significant study results.

In order to keep a consistent theme with our work and more recent work, and allay some of the confusion in defining groups, we have also renamed our groups to be no-FOG, OFF-FOG (freezing in only the OFF levodopa state), ONOFF-FOG (freezing in both the OFF and ON levodopa states).

Finally, since the ONOFF-FOG group had notably higher variances on gait features than OFF-FOG peers, we have added sample size considerations for those who may further study how ONOFF-FOG and OFF-FOG patients differ on gait features and have tried to clarify the reporting of the results to make our points clearer.

ABSTRACT
In the Objective statement, I recommend revising “motor” to “gross motor”, given that gait is a motor function but mentioned separately.

Response: We have made the suggested correction.

In the final sentence of the Results section, please revise “Variability” to “Gait variability” (or, ideally, a more specific gait variability metric – e.g., step length variability).

Response: We thank the reviewer for this suggestion. We have revised this sentence to state “Inter-participant variability in spatiotemporal gait features…” to more accurately reflect our data. Hopefully this rewording more clearly indicates the differences in variability among the FOG groups applies to several of the gait features.

In the Conclusions statement, I recommend revising or omitting the second sentence. This manuscript provides primarily descriptive information about the different subgroups of persons with PD and does not provide causal evidence of factors that may drive levodopa-resistant FoG in PD.

Response: we have reworded this statement to indicate more the descriptive nature as suggested. *“Our results suggest that multifactorial deficits may lead to ONOFF-FOG development.”*

INTRODUCTION
The introduction is quite short and provides only a limited background of information regarding FoG in PD. There are significant bodies of literature investigating the motor response to levodopa in persons with PD and FoG that could help the reader to understand the direction and hypotheses of the study. As currently written, the introduction lacks focused hypotheses about what motor and non-motor features may be expected to differ among the groups of interest and why. Such hypotheses could be formed logically with a more thorough background of proposed mechanisms of both the motor response to levodopa and development of FoG in PD. What might we expect to differ between persons with and without levodopa-resistant FoG and why?

While I agree with the authors in that I am also not familiar with any studies that have directly compared persons with PD and levodopa-resistant FoG to those with levodopa-responsive FoG, there have been studies contrasting persons with PD and levodopa-resistant FoG against a more general population of persons with PD (e.g., Ferraye et al., Eur Neurol, 2013). These studies are relevant but currently omitted from the background.

Response: We thank the reviewer for these comments and have expanded on the Introduction section adding the recommended manuscripts.

METHODS
Given the stated goal of the study (identifying differences between persons with levodopa-resistant/levodopa-responsive FoG), it is not clear why a no-FOG group was included.

Response: We included a noFOG group as prior work has focused on the difference between noFOG and FOG in general and we wanted to ensure that in the patient populations we have, the noFOG/OFF-FOG changes served as a control of sorts when comparing the ONOFF-FOG changes. We have tried to clarify this in the manuscript as below:

*“The differences between measured gross motor, gait and non-motor features between the noFOG to OFF-FOG were used to compare and contrast the differences between the OFF-FOG and ONOFF-FOG groups.”*

What was the reason for excluding frequent fallers? I understand that there could be safety concerns, but one would imagine that procedures could be put in place to minimize fall risk. It seems like omitting frequent fallers would exclude many potential participants particularly within the ON-FOG group.

Response: We thank the reviewer for raising this point. We only excluded people who had a fall frequency of more than 1 fall per day which is a very high fall frequency and significantly increases the risk of participation in the study and the approval process for the institutional review board. We felt that this was not unreasonable. While it may limit the more severe ONOFF-FOG subjects as noted, the frequency of falls in our subjects was still on the order of 8+/-18 falls per month in the ONOFF FOG group which clinically would still be regarded as “frequent fallers”.

This qualifier was added to the text as below:

*“People with a very high fall frequency of greater than 1 fall per day…”*

This fall frequency in each group is also shown in Table 1.

Did all participants take their medications at the same time relative to the start time of their participation in the study (e.g., one hour prior to participation)?

Response: We thank the reviewer for raising this question. As we did not do an ON/OFF evaluation, subjects were assessed when they came in for their research appointment. The dose of levodopa taken was previously listed in Table 1 and the time from last dose of levodopa has been added to Table 1. We have also added a line in the methods section:

*“All participants were evaluated in the levodopa medicated or ON-levodopa state. For the assessments in the study, they were allowed to take their regular medication doses, including levodopa doses, as per their normal dosing schedule. The dose and time from last levodopa dose were documented during the study visit and reported in Table 2*.”

It would be helpful to define the gait parameters “foot-strike-length” and “ambulation time”.

Response: We apologize for this oversight. We have clarified the definitions of all these gait variables by adding an additional table, now Table 1.

Line 84 – “were enrolled” is repeated.

Response: We have corrected this typographical error.

Line 141 – what is meant by “low, middle, and high values of motor UPDRS”?

Response: we have expanded on these definitions as requested. We now specify these values and where they come from.

RESULTS
Several of the results sections are reported very descriptively without statistics to support claims made. This makes interpretation of the results challenging because it is not clear which comparisons resulted in statistically significant differences vs. which comparisons are being made qualitatively. Please include results from your statistical analyses throughout this section.

Response: We agree that the results for the demographics and motor metrics (Table 2) for the 3 FOG groups are descriptive. We did this on purpose because we had no hypotheses or interest in statistically comparing these groups. This approach adheres to good statistical practice: limiting statistical modeling and tests to those outcomes for which statistical inference was planned. For the Table 2 metrics, we do provide enough of the summary statistics so that a reader who wants to statistically compare the groups can do so. For outcomes in Table 3, we should have indicated which results were statistically significant because we had planned to compare among the groups. We have now indicated those results that were statistically significant. For the gait kinematics, hopefully the original wording is clear about which results are statistically significant (OFF vs No) and those that were not (OFF vs ON); we have now revised the text in that subsection.

Please revise the headings in Figure 3 to read “affected” rather than “effected”.

Response: we thank the reviewer for noting this typographical error and it has been corrected.

DISCUSSION
In line with the comment above regarding the results, it is difficult to comment on the discussion given that it is not clear which results were statistically significant.

Response: Hopefully the revised wording of our results make it easier to see the support for statements we make in the Discussion.

I found the contextualization of the findings within previously proposed models of FoG to be challenging to follow. It was not clear to me how comparisons being made between two populations that both experience FoG (levodopa-responsive FoG and levodopa-resistant FoG) provide evidence for or against models of FoG development; it would seem that comparisons between populations with and without FoG would be more insightful in testing these models.

Response: We are trying to show how our results on OFF-FOG and ONOFF-FOG PD patients lends support for features of some of the previously proposed models for FOG development. These are both subtypes of FOG, and in the data used for development of the models discussed, were usually lumped together into a single FOG-group. If we combine our ONOFF and OFF-FOG groups to just discuss no-FOG vs FOG this does not add to the story that already exists, while our data is novel in providing all these features studied in this population of 105 people with PD.

Reviewer #2: INTRODUCTION:
• Incorrect use of the word mechanistic line 71

Response: we have removed the word mechanistic as requested.

• Line 72, are non-motor features not objective? Clarify

Response: We apologize, but we used the term objective in regards to both gait and non-motor features. We have corrected our wording as below:

*“The goal of our study was to identify an objectively quantifiable feature set from gait, gross-motor and non-motor assessments that distinguish between these two FOG subtypes.”*

• Should discuss prior work on levodopa induced FOG, and pseudo-ON FOG, as well as work on cognitive differences between pharmacologic subtypes.

Response: We thank the reviewer for this suggestion and we have added a description of these studies to the introduction as requested.

• Provide reference for the assertion that FOG is initially dopa-responsive then becomes unresponsive

Response: In an autopsy cohort previously published by the corresponding author (Virmani), the group that developed ONOFF-FOG did so approximately 10 years after they developed OFF-FOG. This reference has been added to the introduction.

METHODOLOGY
• Clarify criteria for non-freezers. Did they have to have a 0 on item one of the fogq?

Response: all non-freezers had 0 on item 3 of the FOG-Q. This has been clarified in the text.

• Why was the nfogq not used? If not, this is a limitation of the study and should be mentioned. The original version had several limitations which is why the new version was introduced.

Response: We agree with the reviewer that the FOG-Q had limitations, especially in that the first 2 items ask about gait in general rather than freezing. However item 3 on the FOG-Q is equivalent to item 1 of the N-FOGQ. Just as the NFOGQ demonstrates in a video freezing episodes, all participants in our study were demonstrated freezing before answering item 3 of the FOG-Q.

• classification of ON FOG is unclear and confusing as it is written, please rewrite sentence beginning with “freezers…” in line 93

o it appears you are including levodopa induced FOG as ON FOG
o discussion regarding multiple classifications of dopa response is critical here. Some groups will define ON FOG as dopa induced FOG, others as any FOG in the ON state, and others as FOG that does not respond to dopamine, it looks like all of these were included in this group making it quite heterogenous
o OFF FOG group may also classified based on its dopa response

Response: we thank the reviewer from bringing these multiple points regarding classification of the groups. We have tried to clarify our descriptions of the three groups of participants and to help make the groups clearer, we also renamed our groups based on what appears to be an emerging consensus of no-FOG, OFF-FOG (freezing in only the OFF levodopa state), ONOFF-FOG (freezing in both the OFF and ON levodopa states). We also added a line to explain that none of the participants had what was defined by Espay et al. as ON-FOG (freezing in only the ON-state and not the OFF-state). Previous studies also suggested that pure ON-state but not OFF-state freezers are not common (Amboni et al., McKay et al.).

Item 3 of the FOG-Q is equivalent to Item 1 of the N-FOGQ. The main difference from the N-FOGQ to FOG-Q is the video showing people with different types of freezing. The examiner (TV) a movement disorders trained neurologist demonstrated the freezing phenomena to the participants when documenting freezing.

Also as noted in the methods section TV (a movement disorders neurologist) had been following these participants in his clinic and was aware of their status. This has been clarified further in the methods section.

• There was no evaluation of objective gait parameters in the OFF state, major limitation

Response: we thank the reviewer for raising this point and we have noted this in the limitations section of the discussion and acknowledge that such a study would have added additional information. However the goal of this study was not to look at the levodopa responsiveness in these features. Since the primary interest was the OFF vs ONOFF groups, the differences that persist in the levodopa medicated state should theoretically be of greater interest in classifying the two groups. We have added a line to the discussion to make this point.

*“However, since the OFF-FOG group has improved freezing in the ON-levodopa state while the ONOFF-FOG group still has freezing in the ON-levodopa state, the differences in spatiotemporal gait features that remain in the ON-levodopa state are of primary interest.”*

• It looks like there was no adjustment for multiple comparisons

Response: We now speak to this issue at the end of the Results section. We estimate the number of false discoveries among the 80 comparisons we make in Figures 1, 2, Supplementary Figure 1, and Table 3.

RESULTS
• Please indicate on table 1, which differences were statistically significant

Response: We purposefully provided only summary statistics in the old table 1, now Table 2. Comparing demographics among the groups was not a study objective; i.e., we had no hypotheses regarding these characteristics. As noted in our response to Reviewer #1, this approach adheres to good statistical practice: limiting statistical modeling and tests to those outcomes for which statistical inference was planned. However, for the Table 2 metrics, we do provide enough of the summary statistics so that a reader who wants to statistically compare the groups can do so. Finally, we note that if we were to statistically compare among the groups for these Table 2 characteristics, it would add to the multiple comparison issue Reviewer #2 notes above. For these reasons, we much prefer to leave Table 2 as is.

• Line 184, clarify what evidence there was of any differences

Response: The sentence has been clarified as follows:

“*While apathy scores on the AES were greater in OFF-FOG participants by 3.4 points, no difference (i.e., a difference of 0) was also plausible.”*

• Table 2 needs to be clarified, label specifically what factors are statistically significantly different, not just state that CI’s that don’t include zero are significant, there is no legend to explain what the bolded entries represent

Response: We apologize that the reviewer missed the table title which stated ““CIs not including 0 are statistically significant at p<0.05 **and indicated in bold font***.* We have moved this statement to the Legend as suggested.

• Cannot report “statistically significant” differences if did not correct for multiple comparisons

Response: We now report positive False Discovery Rates, which alerts the reader to the possibility that some results found to be statistically significant may be Type I errors.

• Should not report non statistically significant differences even if there were trends that were not significant due to variance

Response: Unfortunately, we do not agree with the reviewer on this critique for the following reasons. Suppressing planned results that were not statistically significant suppresses the advance of science. So that the non-significant results can be useful, we provided the effect sizes *and* measures of how precisely those effect sizes are known (e.g., confidence intervals). These pieces of statistical information are useful for future studies. Reporting these pieces of statistical information for comparisons that are not statistically significant is good statistical practice. Looking carefully at the data in the figures, one can see that the directionality of the changes is different between the ONOFF- to OFF-FOG vs OFF-FOG to no-FOG comparisons and is therefore an important finding. We therefore have left these few lines of results in the text of the manuscript to guide readers to information we feel is important, that would otherwise be missed if the data were in figures or tables alone.

DISCUSSION
• “In FOG participants with approximately the same disease duration, and age at onset, ON-FOG participants had greater cognitive dysfunction, greater motor disease severity, and lower quality of life than OFF-FOG participants. “ All of this is well known and previously reported. Authors should acknowledge this is well known, and reference prior work in the initial paragraph.

Response: We had referenced prior work in the second paragraph of the discussion, but as requested have added references to the first paragraph. We have also expanded on our discussion as suggested and included other previously reported features that can be compared to our report.

• I see no value in reported and much less discussing non significant trends.

Response: While the trends may be statistically non-significant, the effect sizes were large and therefore we felt worth reporting. We now use our results to inform potential future studies about sample sizes needed to find statistical evidence of differences between OFF-FOG and ONOFF-FOG populations. We believe this sort of information can be useful to those planning future investigations of these two populations.

• Sentence starting in line 278 needs a reference

Response: We have added an overview reference to what was previously line 278. We have also added a line drawing the readers attention to specific studies that are used in the subsequent paragraphs from work on continuous gait.

• It does not follow that based on the fact that there are inter episodic gait changes in FOG there are four models of FOG. There are many other models than those proposed in 2013.

Response: We thank the reviewer for this comment and have edited our sentence accordingly.

• I don’t see how the discussion of these 4 models is helpful in explaining the findings of the work presented here.

Response: We chose to discuss these 4 models as they cover a wide range of suggested changes between the no-FOG and FOG groups. We therefore wanted to place our results for the OFFON-OFF comparison in the context of the no-OFF models. We have attempted to clarify our reasoning in the discussion.

• I suggest only statistically significant findings between groups that survive multiple comparisons be reported and discussed.

Response: Hopefully the reviewer will be satisfied with our addition of False Discovery Rates and the power analyses we now provide.

• Specifically there is no discussion of the findings of figure 1 which imply that the changes that are seen, if they survive multiple comparisons are more likely due to overall motor decline that FOG itelf

Response: we apologize if this was not clear to the reviewer. We have actually discussed the results of Figure 1 as it pertained to the threshold model we just did not label it as Figure 1. We copied below this discussion for the reviewer to be able to access it more easily.

Line 412: *In our study, mean stance phase and total double support, and the CV of foot-strike length in ONOFF-FOG patients were all greater than in OFF-FOG patients by margins that exceeded the confirmed increases found in the same features when comparing OFF-FOG to no-FOG patients (Figure 1). Because standard deviations of these features in ONOFF-FOG patients were over 2 times that in OFF-FOG patients, coupled with relatively small group sizes (26 ONOFF-FOG and 36 OFF-FOG patients), our confidence in the applicability of this model is modest.*

Reviewer #3: Manuscript: Levodopa ON-state Freezing of Gait: Defining Gait and Non-motor Phenotype

This is an excellent contribution to freezing of gait in Parkinson’s disease, both in the OFF and ON states. The additional data on non-motor features distinguishing these subtypes further enhances this study.

Response: we thank the reviewer for taking the time to review our manuscript and for their enthusiasm for our findings.

Overall significance of the paper:

This study demonstrated that ON freezers had greater disease severity, cognitive deficits, and lower quality of life as compared to OFF freezers with similar disease duration.

The authors acknowledged a limitation in not exploring the freezing episodes themselves in detail, in addition to the spatiotemporal indices pre- and post- freezing. This would be a useful next step, as it may yield further distinguishing features between these 2 subgroups.

Response: we agree with the reviewer that this would be the next step to take with our recorded data. We are in the process of developing the algorithms that will help define the freezing episodes from the foot pressure traces provided in PKMAS.

In addition, a future study utilizing 3D motion capture (in this cohort or perhaps collaborating with another institute with such data) would provide further data points, with particular attention to 3D kinematics of arms (i.e. arm-swing), leg swing, and heel/foot strike.

Response: Unfortunately, we do not currently have 3D motion capture in our gait lab, but would be more than happy to collaborate with other centers and possibly obtain common equipment. We have the ability to analyze arm swing from accelerometer recordings in the future.

Other:

Page 4: Line 84 – Duplicate “were enrolled.” Please delete one.

Response: We thank the reviewer for noting this discrepancy and we have corrected this typographical error.
