# Supplementary material for: Levodopa ONOFF-state freezing of gait: Defining the gait and non-motor phenotype
Source: PLoS One. 2022 Jun 2;17(6):e0269227. doi: 10.1371/journal.pone.0269227 (PMC9162361; doi:10.1371/journal.pone.0269227)
Supplement: Attachment — Submitted filename: PONE-D-21-16241R1 - Response to Reviewers v4.docx [file pone.0269227.s008.docx]

Response to reviewers (PONE-D-21-16241R1)

Levodopa ONOFF-state Freezing of Gait: Defining the Gait and Non-motor Phenotype

PLOS ONE

We thank the reviewers for their review of our paper and the reviewers and editors for giving us an opportunity to revise our manuscript. We believe that the manuscript is improved with these changes. We have responded by modifying the manuscript and we also respond item by item to the comments below. We hope that the manuscript will be acceptable now to publication.

==============================
As you can see, the reviewers are still divided about your article. I urge you to respond to all comments carefully. And especially adjust the text about aspects of the statistical procedures and stick only to what has been tested and evaluated quantitatively. For ease of review, please respond to ALL comments, indicating in the response letter where changes have been made (lines and pages) in the manuscript and even copying and pasting these parts of the manuscript into the response letter.
==============================

Author response: We understand that only Reviewer #1 had concerns about appropriate and rigorous statistical analyses, but that the concern was only with our statistical *reporting* in the manuscript, and not with the appropriateness or rigor of the analyses. We think we better understand what the concern was, and try to address it.

Reviewer #1: The authors have partially addressed the prior comments. The statistical reporting remains an issue. If the authors choose not to perform statistical analyses on certain aspects of the data, they should refrain from interpreting the data; that is, if demographic data are included to provide purely descriptive information about each group, the authors should not make statements such as "were not notably dissimilar" and "clearly the highest" without some type of quantitative statistical analysis to substantiate these claims. Certainly, the reader can see the data and ascertain for themselves which numbers are larger than others without additional need for interpretation if there are no statistical results to report.

Author Response: First, thank you for taking the time to review our revision. Regarding your critique above, I (RDL) think I “get it” now. In the paragraph where we summarize demographic and baseline attributes in Table 2, we now refrain from using subjective statements about differences among the three groups.

“Demographics of the PD patients enrolled are shown in Table 2. We did not statistically compare these characteristics among the three groups as we had no hypotheses necessitating such tests. Ages at motor onset and duration of disease did not differ by more than a standard deviation (SD) among FOG groups. Consequently, ages at enrollment also did not differ by more than a standard deviation. Regarding initial symptoms at motor onset, though the ONOFF-FOG group had the highest percentage of patients whose first symptom was in gait, over a third of ONOFF-FOG patients reported rest tremor as their initial symptoms. Hoehn & Yahr scores in the ONOFF-FOG group were higher than the other two groups by about 1½ standard deviations or more, and fall frequencies in the ONOFF-FOG group were over 7 times that in the other two groups.”

Table 3 provides indicators of statistical significance but does not provide specific test statistics or p-values (as are provided for the gait data in the S1 Table).

Author response: We now include the test statistics and *p-*values in Table 3 as requested. We have updated the caption of Table 3 to read as follows.

**Table 3.** Means (and 95% CIs) for motor and non-motor features, adjusted for sex, age at enrollment, and disease duration. The differences in means (and 95% CIs) between the OFF and ONOFF-FOG groups are also provided, with the test statistics and *p*-values. The CIs for differences in means that do not contain 0 are in bold font.

Reviewer #2: The authors present a well written report of a comparative study between PD patients with freezing exclusively in the OFF state and those that have FOG in the ON and in the OFF state. They collect data on multiple domains to replicate prior work, and focus on reporting of quantitative gait findings that may separate the groups. While I do agree that quantitative measurements of gait, outside the FOG episode is of value in FOG, conceptually, there is a flaw in the premise that there would be phenomenological differences in based on dopa-response. Largely, this study fails to find significant behavioral differences in the recorded spatiotemporal parameters between OFF and ONOFF FOG, which is the main premise of the study, and the authors attribute to variability. The differences that are observed in cognition, quality of life, etc., can largely be explained by a greater disease severity. Moreover, these findings have been previously reported, particularly in cognition, with more thorough analyses, and this study did not add significantly to these findings. The discussion is well written and extensive, however, I do not see how the discussion of models of FOG is relevant to the findings of the study. I do agree on their depiction of the literature, the models, and dopa-response in FOG as a continuum, but again, I do not see how the data they present supports these assertions. There is a very transparent presentation of the data, and their analysis, and given the data presented, I believe their statistical approach, whereby FDR and effect sizes are presented, is justified.

Author response: Thank you for reviewing our last revision; we know it takes time to do so. We are glad that Reviewer #2 recognizes our effort to be transparent with our treatment of the data and results. However, Reviewer #2 notes that we have a “flaw in the premise that there would be phenomenological differences based on dopa-response.” The lack of evidence that differences in the mechanisms of progression exist between those with OFF-FOG and those with OFF-ON FOG does not provide evidence that the mechanisms are the same; that is, “absence of evidence is not evidence of absence.” The results we present can be useful to other researchers in that (*i*) they may gather larger samples to increase power for detecting differences having magnitudes in line with what we found, or (*ii*) they may decide not to pursue this line of research. Either way, this work is useful.

Our previous published results (Glover et al. 2020) show that people with noFOG and OFF-FOG have different progression in gait parameters. So one could hypothesis as the reviewer is suggesting that they are the same, just a matter of severity and if everyone lived long enough then everyone would develop levodopa resistant freezing. However, it has also been proposed that OFF-FOG, ONOFF-FOG and ON-FOG are distinct phenotypes (see Lucas McKay et al. 2019, Introduction for review). The more information on this problem that is published will ultimately help guide us to the answer.

Finally, we have also revised the discussion as requested to focus less on the models themselves and more on the results. Hopefully these changes make the discussion more acceptable.

Reviewer #3: I agree with Reviewer #1 regarding renaming the various FOG subgroups as it was difficult to follow the data at times without this added detail.

Author response: Thank you for the review of the revision. We also are glad Reviewer #1 made the suggestion.
