# Supplementary material for: Levodopa ONOFF-state freezing of gait: Defining the gait and non-motor phenotype
Source: PLoS One. 2022 Jun 2;17(6):e0269227. doi: 10.1371/journal.pone.0269227 (PMC9162361; doi:10.1371/journal.pone.0269227)
Supplement: Attachment — Submitted filename: Landes et al. - Response to reviewers revision 3-v1.docx [file pone.0269227.s009.docx]

Response to reviewers:

PONE-D-21-16241R2

We thank the reviewers for taking the time to review our manuscript. We have responded to the reviewers suggestions in an itemized format below and also made edits to our manuscript as recommended.

The authors performed a hard work replying all questions raised by the reviewers.

Author response: we thank the reviewer for their appreciation of our efforts on our prior revision.

I have just a couple of minor comments. Firstly, I miss a discussion in the discussion (particularly in lines 260-280) benchmarking the results with high level o evidence on gait biomechanics of PD (PMID: 33436993).

Author response: we thank the reviewer for referring us to this manuscript. We have added a discussion with respect to the provided reference in the discussion section as shown below.

“Continuous gait abnormalities have been well documented in PD patients, and a recent meta-analysis showed consistent reduction in stride-length, swing-time and hip excursion compared to healthy controls [25].”

Secondly, not compulsory, but as a suggestion, try to reply these questions: how do your paper may help to health professionals to prescribe exercise for PD from your findings? (PMID: 34803726 ).

Author response: we thank the reviewer for suggesting this. We have added a few sentences to the discussion suggesting that exercise and therapy should be considered.

*“Whatever the case, exercise has been shown to help possibly with decreasing disease progression and helping acutely with improving gait, balance and FOG and should be consistently recommended to all PD patients [53-55].”*

We have also added a discussion with respect to APAs and gait initiation and freezing as this topic was in both this article and the other article referred to below.

*“Additionally, anticipatory postural adjustments (APAs) have been previously reported to contribute to gait initiation deficits in PD patients [44] and FOG patients [45-47] and further work is needed to determine how these may defer between ONOFF- and OFF-FOG patients and whether impaired APAs impact the extent of freezing duration in the different groups.”*

Are there some 'bridges' between akinetic-rigid and hyperkinetic PD and your findings? PMID: 33517030, PMID: 34803726.

Author response: we thank the reviewer for this question. It is an interesting one as it is suggested that akinetic-rigid PD is either the same phenotype as FOG or a predictor of FOG. 100% of our ONOFF-FOG cohort were of the akinetic rigid phenotype at the time of visit. Additionally it has been reported that people can evolve from a tremor-predominant to akinetic rigid phenotype over time as tremor is more responsive to levodopa.

*“In our cohort, there was an increasing percentage of people meeting the PIGD phenotype designation going from noFOG to OFF-FOG to ONOFF-FOG. It remains unclear whether these two phenotypes, PIGD and FOG are independent risk factors for one another, or possibly the same phenotype. Similar to the increasing incidence of FOG from 7% in early disease [1] to 92% at death [2], the PIGD phenotype has also been reported to increase with disease duration with one cohort study reporting 54% PIGD participants at initial visit, 73% at 4-year follow-up and 88% at 8-year follow-up [51], albeit limited by drop-out over this period in the total population due to deaths in the cohort. Disease features associated with the PIGD phenotype are also similar to FOG including longer disease duration, greater disease severity, lower cognitive scores, impaired postural adjustments and faster disease progression [51-54]. We previously showed that FOG participants show faster spatiotemporal gait decline than noFOG [55]. Clinically OFF-FOG can respond well to levodopa and when motor-fluctuations are not occurring in early PIGD, freezing may go unwitnessed either in the form of micro-freezes, or levodopa responsive freezing. Further work is needed in larger longitudinal cohorts to explore these two different possibilities.”*
